# Kaempferol Promotes Apoptosis While Inhibiting Cell Proliferation via Androgen-Dependent Pathway and Suppressing Vasculogenic Mimicry and Invasion in Prostate Cancer

**DOI:** 10.1155/2019/1907698

**Published:** 2019-12-01

**Authors:** Jun Da, Mingxi Xu, Yiwei Wang, Wenfeng Li, Mujun Lu, Zhong Wang

**Affiliations:** ^1^Department of Urology, Shanghai Ninth People's Hospital, Shanghai Jiao Tong University School of Medicine, Shanghai, China; ^2^Department of Urology, Shanghai Renji Hospital, Shanghai Jiao Tong University School of Medicine, Shanghai, China

## Abstract

Kaempferol is a well-known natural flavonol reported to be a potential treatment for multiple cancers. In this study, we demonstrated that cell growth of androgen-sensitive LNCaP cells could be inhibited 33% by 5 *μ*M kaempferol, around 60% by 10 *μ*M kaempferol, and almost 100% by 15 *μ*M kaempferol. Also, kaempferol showed relatively limited effect on PC-3 cells and nonmalignant RWPE-1 cells. In the presence of DHT, the IC_50_ for kaempferol was 28.8 ± 1.5 *μ*M in LNCaP cells, 58.3 ± 3.5 *μ*M in PC-3 cells, and 69.1 ± 1.2 *μ*M in RWPE-1 cells, respectively. Kaempferol promotes apoptosis of LNCaP cells in a dose-dependent manner in the presence of dihydrotestosterone (DHT). Then, luciferase assay data showed that kaempferol could inhibit the activation of androgen receptors induced by DHT significantly. The downstream targets of androgen receptors, such as PSA, TMPRSS2, and TMEPA1, were found decreased in the presence of kaempferol in qPCR data. It was then confirmed that the protein level of PSA was decreased. Kaempferol inhibits AR protein expression and nuclear accumulation. Kaempferol suppressed vasculogenic mimicry of PC-3 cells in an in vitro study. In conclusion, kaempferol is a promising therapeutic candidate for treatment of prostate cancer, where the androgen signaling pathway as well as vasculogenic mimicry are involved.

## 1. Introduction

Prostate cancer is the second most common cancer and the fifth leading cause of cancer-related death in males globally. It occurs more commonly in the developed world and is the most common cancer in men worldwide [1]. The combination of surgery, hormone therapy, radiation therapy, or chemotherapy may be much more common treatments for prostate cancer currently. The outcomes of treatments for prostate cancer depend on the patients' age and how aggressive as well as extensive the cancer is. 30% to 70% of males over age 60 involved in prostate studies who died from unrelated causes have been found to be suffering from prostate cancer. It remains necessary to find new ways to treat prostate cancer effectively.

Kaempferol is firstly named after a German naturalist Engelbert Kaempfer. It is a natural flavonol, found in a number of fruits and vegetables. Kaempferol is a yellow crystalline solid with a melting point of 276–278°C (529–532°F). It is slightly soluble in water and highly soluble in hot ethanol, ethers, and DMSO. Many reports demonstrated that consuming kaempferol may reduce the risk of various cancers such as colon cancer [2], hepatoma [3], and bladder cancer [4]. Kaempferol has been studied in several kinds of malignant tumors, including malignant tumors of the urology system. As reported, it can inhibit cell growth of renal cancer cells [5, 6], bladder cancer cells [7, 8], and prostate cancer cells [9]. And it is currently under consideration as a possible cancer treatment. The systematic role of kaempferol in prostate cancer remains unclear. In the present study, we explored the effect of kaempferol on cell growth, apoptosis, vasculogenic mimicry, and invasion of different types of prostate cancer cells, and DHT or AR involved in the pathway of kaempferol related to prostate cancer was also investigated.

## 2. Materials and Methods

### 2.1. Cell Lines and Culture

Cell lines of HEK293, LNCaP, PC-3, and RWPE-1 were used in our study. HEK293, LNCaP, and PC-3 were supplied by the Cellbank of Chinese Academy of Sciences (Shanghai, China), and RWPE-1 was purchased from the American Type Culture Collection (ATCC, USA). PC-3 and LNCaP were maintained in RPMI-1640 media with 10% fetal bovine serum and 1% penicillin/streptomycin. RWPE-1, the nonmalignant human prostate cell line, was maintained in keratinocyte serum-free medium (Invitrogen, catalog no. 10724) and supplements (Invitrogen, catalog no. 37000-015). The HEK293 cell line was generated by transformation of human embryonic kidney cells and maintained in Dulbecco's modified Eagle's medium (Life Technologies) supplemented with 10% FBS and antibiotics. All cells were incubated in 37°C with 5% CO_2_.

### 2.2. Luciferase Assays

Refer to the method in our previous studies [10, 11]. In brief, HEK293 and LNCaP cells lacking of functional AR were transfected with wild-type AR expression plasmid and reporter genes such as pSG5-AR, pSG5-PR, pSG5-GR, MMTV-Luc, and pRL-TK-luc for AR for 24 h. Then, the cells were cocultured with various concentrations of kaempferol in the presence or absence of 1 nM dihydrotestosterone (DHT) (Sigma, USA) and/or 5 *μ*M hydroxyflutamide (HF) (Sigma, USA) for 24 h. The cells were harvested and assayed for luciferase activity. Data were expressed as relative luciferase activity normalized to the internal Renilla luciferase control.

The mammalian 2-hybrid assay was performed to determine the AR N-C interaction and AR-AR coregulator interaction. After transfection with pGal4-RE-Luc reporter plasmid, pGal4-ARDBD-LBD (AR DNA-binding domain and ligand-binding domain), pCMX-VP16-AR, or pCMX-VP16-ARA70 for 24 h, HEK293 and LNCaP cells were harvested for dual luciferase assay (Promega, WI).

### 2.3. MTT Assay and IC_50_ Value Determination In Vitro

For MTT assay, LNCaP, PC-3, and RWPE-1 cells were seeded in a 24-well plate at 2500-8000 cells/well. 5 *μ*M, 10 *μ*M, and 15 *μ*M of kaempferol were added to wells with/without 1 nM DHT from day 0 and changed every other day. Thiazolyl blue tetrazolium bromide solution (5 mg/mL, Sigma) was added into each well and incubated in 37°C with 5% CO_2_ for one hour. Samples were collected at days 0, 2, 4, and 6. Then, 300 *μ*L DMSO was added and the O.D. value was detected at 570 nm by a microplate reader (Synergy Mx, BioTek).

Cytotoxicity assay was performed according to the protocol reported in a previous study [12]. To determine the IC_50_ value, 1.0 × 10^6^ LNCaP cells, 5.0 × 10^5^ RWPE-1 cells, and 1.0 × 10^6^ PC-3 cells were plated in triplicate in 24-well culture plates. The cells were incubated with serial concentrations of kaempferol for 2 days, and cell viability was determined in triplicate by the MTT assay. Kaempferol medium (no cells) was included as controls. Kaempferol-treated cells were compared to untreated cell control wells. The IC_50_ value was analyzed with the program CompuSyn (Developer).

The MTT assay was performed to evaluate cell growth, cells in which were expected to grow for 6 days, as well as cells in IC_50_ value determination were expected to grow for 2 days. Therefore, we used two different culture conditions for the MTT assay and IC_50_ value determination.

### 2.4. Cell Apoptosis Assay

Cell apoptosis was determined by flow cytometry using the Annexin V-APC Apoptosis Detection Kit (Sungene Biotech, Shanghai, China). After being treated with DHT and/or kaempferol for 4 days, the harvested cells were then washed in PBS followed by incubation with Annexin V and PI (propidium iodide) in a binding buffer in the dark at room temperature for 30 min. The FACSCalibur Flow Cytometer (BD Biosciences, USA) was used to analyze the stained cells.

### 2.5. Vasculogenic Mimicry Assay In Vitro

PC-3 cells were planted in the Matrigel-coated 96-well plates with a density of 0.2 × 10^5^ cells/well. For treatment, different concentrations of kaempferol were added to the cells. After treatment with kaempferol for 24 h, photographs of cells were taken and the number of vasculogenic mimicry formation was counted.

### 2.6. Invasion Assay by Transwell

For invasion assay, Transwell plates (Corning, USA) were used. Briefly, 1 × 10^5^ cells/well treated with different concentrations of kaempferol in 200 *μ*L RPMI-1640 without FBS were seeded in the upper inserts which were coated with Matrigel. And then, 500 *μ*L RPMI-1640 supplemented with 10% FBS was added in the lower wells. After incubation for 24 h, the cells on the top of the upper inserts were scraped, then the invaded cells on the bottom of the inserts were fixed using 4% paraformaldehyde and stained by crystal violet. The numbers of invaded cells were quantified using OD at 570 nm of cells from a microplate reader.

### 2.7. RNA Extraction and Quantitative Real-Time PCR Analysis

RNA extraction and quantitative real-time PCR analysis were performed according to our previous studies [10, 11]. Briefly, after RNA extraction from cells and reverse transcription, quantitative real-time PCR analyses were performed using the SYBR Green PCR Master Mix Kit (Perkin Elmer, MA, USA) according to the manufacturer's instructions. The amplification system and primers for AR, PSA, TMPRSS2, TMEPA1, and *β*-actin normalization control were identical to previous studies [10, 11].

### 2.8. Western Blot Analysis

Cells were lysed in RIPA buffer (50 mM Tris-HCl/pH 7.4, 1% NP-40, 150 mM NaCl, 1 mM EDTA, 1 mM PMSF, 1 mM Na_3_VO_4_, 1 mM NaF, 1 mM okadaic acid, and 1 mg/mL aprotinin, leupeptin, and pepstatin). Individual samples (30-60 *μ*g protein) were prepared for electrophoresis run on 5–8% SDS/PAGE gel and then transferred onto PVDF membranes (Minipore). After blocking the membranes with 5% fat-free milk in TBST (50 mM Tris/pH 7.5, containing 0.15 M NaCl and 0.05% Tween-20) for 1 h at room temperature, the blots were probed with primary anti-AR (N-20) (Santa Cruz Biotechnology) and anti-PSA (C-19) (Santa Cruz Biotechnology) antibodies with dilutions of 1 : 500 to 1 : 1,000 overnight at 4°C. After washing, the secondary antibody (rabbit anti-goat (IgG 1 : 5,000 dilution; Santa Cruz Biotechnology) or rabbit anti-mouse IgG (1 : 5,000 dilution; Pierce Chemical, Rockford, IL)) was used at room temperature for 1 h. Immunoblot analysis was performed with horseradish peroxidase-conjugated anti-rabbit or anti-mouse IgG antibodies using enhanced chemiluminescence reagents for western blotting detection (Amersham Biosciences).

### 2.9. Immunofluorescence

For the immunofluorescence staining of AR in LNCaP cells, cells were plated onto glass coverslips. After being treated with DHT and/or kaempferol, cells were fixed and permeabilized with cold methanol for 15 min at −20°C, then washed and followed by being blocked with 5% normal goat serum and 0.3% Triton X-100 for 1 h at room temperature. Cells on coverslips were then incubated with anti-AR antibodies, 1 : 500 dilution (Cell Signaling Technology) at 4°C overnight, and then incubated with secondary antibody of Alexa Fluor 647 goat anti-rabbit antibody. DAPI was used to counterstain the nuclei, and the stained cells were visualized using fluorescence microscopy (TCS SP8 Leica, Germany).

### 2.10. Statistical Analysis

Data are presented as mean ± standard deviation (SD). Differences between groups were assessed using Student's *t*-test. *p* < 0.05 is considered statistically significant.

## 3. Results

### 3.1. Kaempferol Inhibits the Growth of AR-Positive Prostate Cancer Cells Much More Than AR-Negative Ones or Nonmalignant Prostate Cells

**Figure 1:**
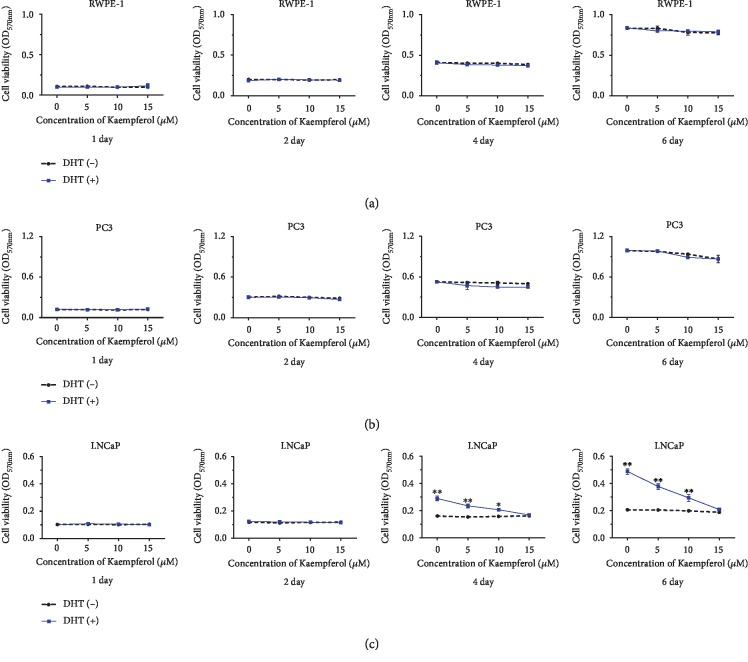
The effect of kaempferol on cell viability of prostate cancer cells. (a, b) Cell growth of RWPE-1 and PC-3 cells was slightly affected by DHT and kaempferol. (c) Kaempferol inhibited cell growth of LNCaP cells up to 33% at 5 *μ*M, around 60% at 10 *μ*M, and almost 100% at 15 *μ*M (p < 0.05) in the presence of 1 nM DHT on days 4 and 6 (*n* = 3). ^∗^*p* < 0.05, ^∗∗^*p* < 0.01 compared with cells without DHT.

The effect of kaempferol on the cell growth of LNCaP, PC-3, and RWPE-1 cells was investigated in our study. It is well known that LNCaP cells were AR-positive cells and PC-3 cells were known as AR-negative cells. RWPE-1 cells were nonmalignant prostate epithelial cells. 1 nM DHT was added to mimic androgen hormone level after castration. Data of MTT assay showed that cell viability of LNCaP decreased 33% by 5 *μ*M kaempferol, about 60% by 10 *μ*M kaempferol, and almost 100% by 15 *μ*M kaempferol (Figure 1(c)) on days 4 and 6. On the contrary, the cell growth of PC-3 and RWPE-1 was not inhibited significantly by the same dosage of kaempferol, regardless of the presence of DHT (Figures 1(a) and 1(b)).

**Figure 2:**
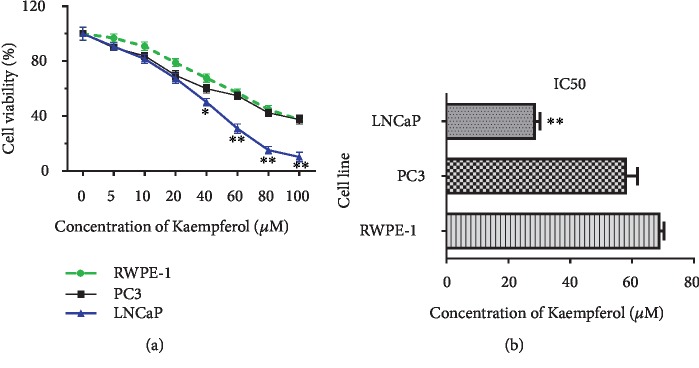
(a, b) IC_50_ of kaempferol with DHT. IC_50_ of kaempferol was 28.8 ± 1.5 *μ*M, 58.3 ± 3.5 *μ*M, and 69.1 ± 1.2 *μ*M in LNCaP, PC-3, and RWPE-1 cells with 1 nM DHT (*n* = 3). ^∗^*p* < 0.05, ^∗∗^*p* < 0.01 compared with RWPE-1.

The half maximal inhibitory concentration (IC_50_) is used to measure the effectiveness of a compound in inhibiting biological or biochemical function. A dose-response curve was used to determine IC_50_ values and examine the effect of different concentrations of antagonist on reversing agonist activity. In the present study, the IC_50_ concentrations for kaempferol on the cells were evaluated by half-inhibition of cell growth at 48 h of kaempferol treatments with 1 nM DHT. In the presence of DHT, the IC_50_ concentrations for kaempferol in LNCaP, PC-3, and RWPE-1 were 28.8 ± 1.5 *μ*M, 58.3 ± 3.5 *μ*M, and 69.1 ± 1.2 *μ*M, respectively (Figures 2(a) and 2(b)).

### 3.2. Kaempferol Promotes the DHT-Dependent Apoptosis of AR-Positive Prostate Cancer Cells

**Figure 3:**
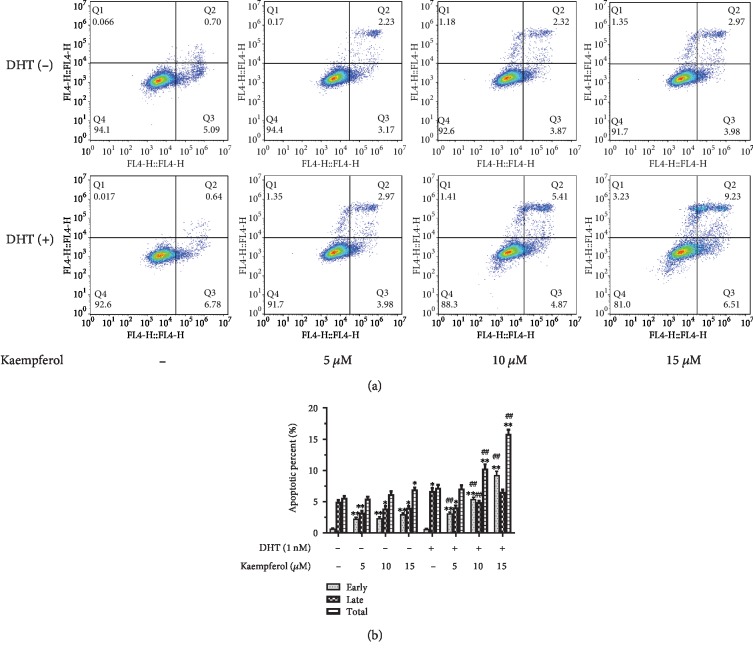
Kaempferol promotes the DHT-dependent apoptosis of AR-positive prostate cancer cells. (a) Apoptosis of LNCaP cells was analyzed using flow cytometry. (b) Kaempferol promotes early and total apoptosis in a dose-dependent manner in the presence of 1 nM DHT (*n* = 3). ^∗^*p* < 0.05, ^∗∗^*p* < 0.01 compared with cells without DHT and kaempferol treatment. ^##^*p* < 0.01 compared with cells with DHT but without kaempferol treatment.

Considering the effect of kaempferol on the cell growth of AR-positive prostate cancer cells significantly compared with that of AR-negative ones or nonmalignant prostate cells, we further evaluated the effect of kaempferol on apoptosis of prostate cancer cells. As shown in Figure 3, kaempferol could promote apoptosis of LNCaP cells, AR-positive prostate cancer cells, in a dose-dependent manner significantly. It was worth noting that the effect of kaempferol on apoptosis of prostate cancer cells is in a DHT-dependent manner.

### 3.3. Kaempferol Specifically Inhibits the DHT-Mediated AR Activation

**Figure 4:**
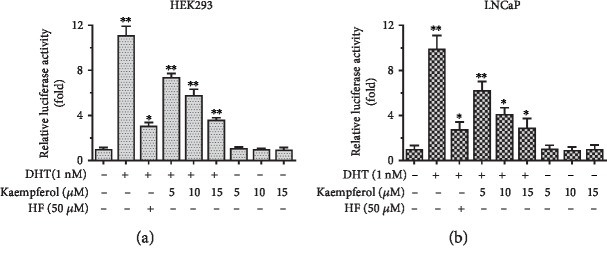
Kaempferol inhibited the AR activation in HEK293 and LNCaP cells. (a) In HEK293 cells, HF inhibited DHT-induced activation of AR by 72.46%. Kaempferol inhibited the AR activation by 33.53% at 5 *μ*M, 47.90% at 10 *μ*M, and 67.66% at 15 *μ*M. (b) In LNCaP cells, HF inhibited DHT-induced activation of AR by 72.13%. Kaempferol inhibited the AR activation by 37.19% at 5 *μ*M, 58.40% at 10 *μ*M, and 70.64% at 15 *μ*M (*n* = 3). ^∗^*p* < 0.05, ^∗∗^*p* < 0.01 compared with cells without kaempferol, DHT, and HF.

We explored whether kaempferol could modulate AR function. At first, the effect of kaempferol on the regulation of AR transactivation activity was evaluated in HEK293 cells. HEK293 cells were transiently transfected with AR and the reporter construct (mouse mammary tumor virus (MMTV)-Luc) containing AR response element (ARE). The relative luciferase activity was measured. As expected, we found that DHT activated AR transactivation and hydroxyflutamide (HF) blocked this activation. Interestingly, kaempferol showed the ability to suppress AR transactivation induced by 1 nM DHT. And this inhibiting effect of kaempferol was dose-dependent as shown in Figure 4(a).

Then, LNCaP cells were used to further confirm the inhibition effect of kaempferol in AR transactivation. AR transactivation was induced by 1 nM DHT and blocked by HF in LNCaP cells followed by the addition of 5 *μ*M, 10 *μ*M, or 15 *μ*M of kaempferol. The results showed that kaempferol could effectively restrain AR activity induced by DHT (Figure 4(b)).

### 3.4. Kaempferol Suppresses AR Target Gene Expression in LNCaP Cells

**Figure 5:**
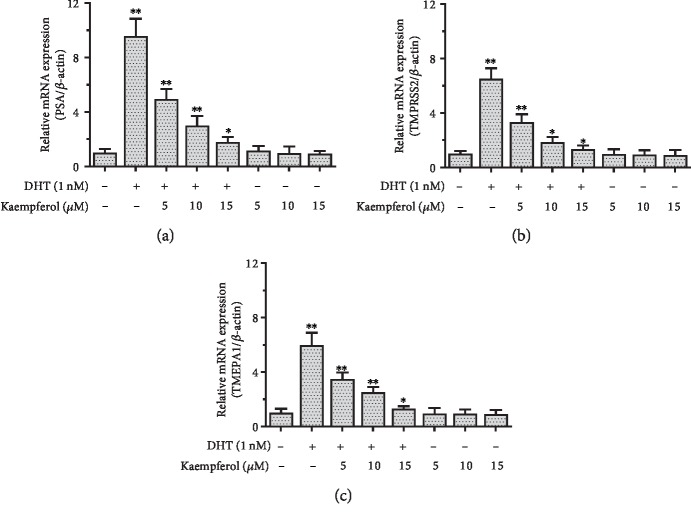
Kaempferol suppresses mRNA expression of AR target gene in LNCaP cells in the presence of DHT. (a) Kaempferol suppresses PSA mRNA expression by 48.25% at 5 *μ*M, 68.62% at 10 *μ*M, and 81.27% at 15 *μ*M. (b) Kaempferol suppresses TMPRSS2 mRNA expression by 49.14% at 5 *μ*M, 71.67% at 10 *μ*M, and 79.48% at 15 *μ*M. (c) Kaempferol suppresses TMEPA1 mRNA expression in qPCR by 41.45% at 5 *μ*M, 58.06% at 10 *μ*M, and 78.12% at 15 *μ*M (*n* = 3). ^∗^*p* < 0.05, ^∗∗^*p* < 0.01 compared with cells without kaempferol and DHT.

To further investigate the ability of kaempferol to regulate the AR downstream genes, we assayed AR target gene expression in AR-positive LNCaP cells. Our data showed that the AR target gene (PSA, TMPRSS2, and TMEPA1) mRNA levels have induced upregulation by 1 nM DHT. Then, 5 *μ*M, 10 *μ*M, and 15 *μ*M kaempferol could effectively suppress the DHT-induced AR target gene expression in LNCaP cells (Figure 5).

### 3.5. Kaempferol Suppresses AR and PSA Protein Expression in LNCaP Cells in the Presence of DHT

**Figure 6:**
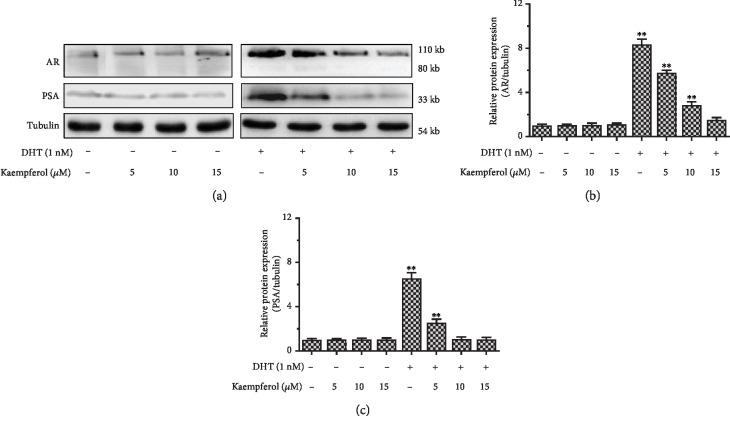
Kaempferol suppressed AR and PSA protein expression in LNCaP cells in the presence of 1 nM DHT. (a) The protein expression of AR and PSA was measured by western blotting. (b) The relative protein expression of AR was quantified by AR blots to Tubulin. (c) The relative protein expression of PSA was quantified by PSA blots to Tubulin (*n* = 3). ^∗∗^*p* < 0.01 compared with cells without DHT and kaempferol treatment.

Our study found that kaempferol inhibited the AR-mediated activity and target gene expression. We then tried to confirm the inhibiting effect of kaempferol on AR and PSA at protein level by western blot. Our data showed that AR and PSA were significantly declined in LNCaP cells treated with 5 *μ*M, 10 *μ*M, and 15 *μ*M kaempferol with the presence of 1 nM DHT (Figure 6). The PSA protein gradually decreased in accordance with increasing kaempferol concentration. Consistent with Figure 4 data, we found that the protein levels of AR and PSA induced by DHT can be inhibited by kaempferol (Figure 6).

### 3.6. Kaempferol Suppresses AR Expression and Nuclear Accumulation in LNCaP Cells in the Presence of DHT

**Figure 7:**
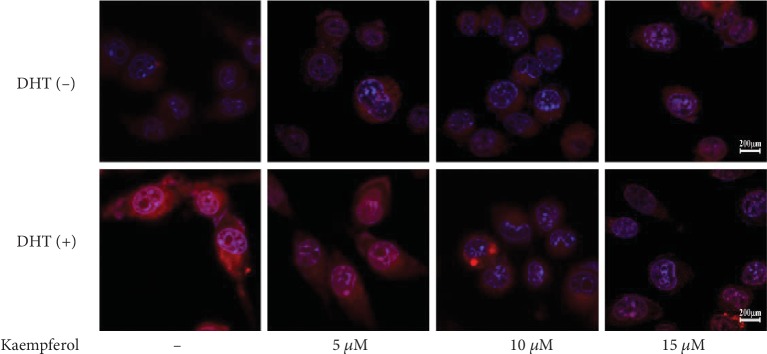
Kaempferol suppresses AR expression and nuclear accumulation in LNCaP cells in the presence of DHT measured using immunofluorescence method. AR protein expression and nuclear accumulation in LNCaP cells were inhibited by kaempferol in a dose-dependent manner in the presence of 1 nM DHT.

To investigate the effect of kaempferol on AR expression and location with the presence of DHT, immunofluorescence assay was performed in our study. As shown in Figure 7, 1 nM DHT upregulated AR expression and induced AR nuclear accumulation which was alleviated and/or reversed by dose-dependent kaempferol at concentrations in the 10-15 *μ*M range.

### 3.7. Kaempferol Suppresses Vasculogenic Mimicry Formation and Invasion in PC-3

**Figure 8:**
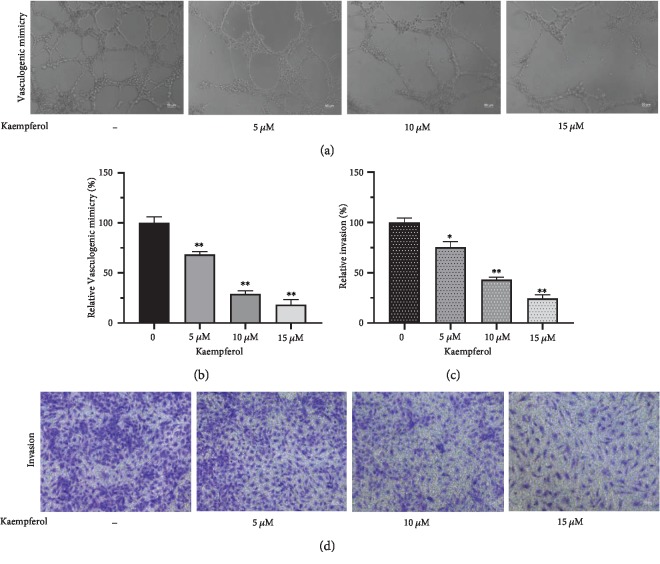
Kaempferol inhibits vasculogenic mimicry formation and invasion in PC-3. Kaempferol inhibited vasculogenic mimicry formation and invasion in PC-3 cells in a dose-dependent manner (5-15 *μ*M) (*n* = 3). ^∗^*p* < 0.05, ^∗∗^*p* < 0.01 compared with cells without kaempferol treatment.

Considering that kaempferol still has effects on cell proliferation in PC-3, we further explored the role of it in vasculogenic mimicry formation and invasive ability of PC-3. As shown in Figures 8(a) and 8(b), kaempferol inhibited vasculogenic mimicry formation in a dose-dependent manner (5-15 *μ*M). The effect of kaempferol on the inhibition of invasive ability of PC-3 (Figures 8(c) and 8(d)) was compliant to that on vasculogenic mimicry.

## 4. Discussion

Prostate cancer has been reported to be one of the most common cancers for male both in incidence rate and morbidity. It is well known that the androgen/AR signaling pathway is crucial for prostate cancer development. So androgen deprivation therapy (ADT) has been well accepted as the first-line treatment for patients with no indications of radical prostatectomy. Except the well-documented flutamide, new chemicals appear to show therapeutic effects on prostate cancer cells. In our previous study [10, 11], Cryptotanshinone and Baicalein, structures of which are similar to DHT, were found to suppress androgen receptor-mediated growth in androgen-dependent prostate cancer cells. The structure of kaempferol, similar to Cryptotanshinone or Baicalein, is very close to DHT too (Figure 1). Moreover, kaempferol is more widely found in fruits and vegetables than Cryptotanshinone or Baicalein. Several studies showed therapeutic effect of kaempferol on different types of cancers, including lung cancer [13], gastric cancer [14], pancreatic cancer [15], breast cancer [16], ovarian epithelial carcinoma [17], renal caner [5], bladder cancer [7, 18], and prostate cancer [9]. Our data confirmed the phenotype that kaempferol inhibited cell growth of AR-positive prostate cancer cell lines, LNCaP. But kaempferol showed limited effects on nonmalignant RWPE-1 and AR-negative PC-3, which was similar to the data reported [9]. Kaempferol showed significant inhibitory effect on prostate cancer cells, while it showed very limited effect on nonmalignant prostate cells. But as kaempferol also showed very limited effect on PC-3 cells, this could imply its limited indication for castration-resistant prostate cancer.

Then, the IC_50_ of kaempferol was evaluated in our lab setting. Our data showed that 48 h treatment of kaempferol with the presence of 1 nM DHT inhibited the proliferation of the LNCaP cells with an IC_50_ value of 28.8 *μ*M, PC-3 58.3 *μ*M, and RWPE-1 69.1 *μ*M, respectively. Due to the use of different kaempferol derivatives, cell culture condition with the addition of DHT to mimic the actual environment in vivo, and time point differences, our result is different from published data [9]. Kaempferol promotes apoptosis of LNCaP cells in a dose-dependent manner at the concentration of 5-15 *μ*M in the presence of 1 nM DHT in our study.

Using HEK293 cells as a tool cell line, our data showed that 1 nM DHT could activate AR downstream signals. As expected, HF could significantly inhibit the DHT-induced AR activation. The addition of kaempferol could inhibit the activation of AR as well. The same phenotype was shown in the LNCaP cell line, which implied that kaempferol could specifically inhibit AR activation induced by androgen. This data showed a confirmative therapeutic potential of kaempferol in prostate cancer. As prostate cancer is well known as an androgen-dependent cancer, this result shows kaempferol as a promising candidate for prostate cancer treatment in hormone-sensitive prostate cancer.

Then, we looked into the changes of androgen downstream signals using kaempferol treatment. In LNCaP cells, the expression of androgen downstream signals, such as PSA, TMPRSS2, and TMEPA1, was significantly decreased after the addition of kaempferol. Further, PSA western blot result confirmed this data at protein level. DHT-induced AR expression and nuclear accumulation were also inhibited by kaempferol in a dose-dependent manner. These data showed that the inhibitory effect of kaempferol on hormone-sensitive prostate cancer cells could be a direct effect of the inhibited activation of androgen receptor and its downstream signals.

Vasculogenic mimicry is known as nonendothelialized vessel-like channels in malignant tumors which could be dependent on to supply blood for tumor growth [19]. As stated in previous reports, vasculogenic mimicry is closely related with metastasis and poor prognosis in many tumors such as melanoma [20, 21], glioma [22, 23], breast cancer [24], and hepatic carcinoma [25]. Vasculogenic mimicry was considered to be associating with epithelial-mesenchymal transition [26] and as a marker of poor prognosis in prostate cancer [27]. We investigated the effect of kaempferol on vasculogenic mimicry of prostate cancer cells in the present study. Considering the ability of tube formation of cells, PC-3 was chosen for vasculogenic mimicry and invasion assay according to a previous study [28]. Our results indicated that kaempferol suppressed vasculogenic mimicry formation and invasive ability in a dose-dependent manner at a concentration of 5 to 15 *μ*M.

It has been reported that kaempferol inhibits LNCaP cell growth via caspase cascade pathway [9]. And cancer cell growth also could be inhibited by kaempferol via inhibition of certain P450 isozyme [29], regulation of MAPK pathway [7, 30], inhibition of PLK1 [31], decreasing levels of IL-6 and TNF (tumor necrosis factor) [32, 33], and inhibition of VEGF (vascular endothelial growth factor) via the ERK-NFkB-cMyc-p21 pathway [34]. From our data, we deduct that the AR signaling pathway could be one of the mechanisms that lead to the therapeutic effect of kaempferol on prostate cancer cells and kaempferol may affect metastasis mediated by vasculogenic mimicry formation; in spite of these, further study remains necessary.

## 5. Conclusion

Kaempferol inhibits cell proliferation and promotes apoptosis of prostate cancer cell lines significantly, especially LNCaP cells, which is an AR-positive prostate cancer cell line, compared with noncancer cells lines, such as RWPE-1. Kaempferol inhibited vasculogenic mimicry formation and invasive ability in a dose-dependent manner. Kaempferol may be a promising candidate for prostate cancer chemical treatment.

## Data Availability

All the figures used to support the findings of this study are included within the article.

## Supplementary Materials

Supplementary Materials (A) Dihydrotestosterone (DHT) which is an androgen. (B) Kaempferol, a natural flavonol widely found from fruits and vegetables. (C) Cryptotanshinone, a major tanshinone isolated from Salvia miltiorrhiza and exhibits multiple activities. (D) Baicalein, a flavonoid originally isolated from the roots of Scutellaria baicalensis Georgi. The structure of kaempferol, Cryptotanshinone, or Baicalein is similar to DHT.

## References

[1] Daniyal M., Siddiqui Z. A., Akram M., Asif H. M., Sultana S., Khan A. (2014). Epidemiology, etiology, diagnosis and treatment of prostate cancer. *Asian Pacific Journal of Cancer Prevention*.

[2] Riahi-Chebbi I., Souid S., Othman H. (2019). The phenolic compound kaempferol overcomes 5-fluorouracil resistance in human resistant LS174 colon cancer cells. *Scientific Reports*.

[3] Kaur P., Robin, Mehta R. G., Singh B., Arora S. (2019). Development of aqueous-based multi-herbal combination using principal component analysis and its functional significance in HepG2 cells. *BMC Complementary and Alternative Medicine*.

[4] Wu P., Meng X., Zheng H. (2018). Kaempferol attenuates ROS-induced hemolysis and the molecular mechanism of its induction of apoptosis on bladder cancer. *Molecules*.

[5] Song W., Dang Q., Xu D. (2014). Kaempferol induces cell cycle arrest and apoptosis in renal cell carcinoma through EGFR/p38 signaling. *Oncology Reports*.

[6] Ahn M. R., Kunimasa K., Kumazawa S. (2009). Correlation between antiangiogenic activity and antioxidant activity of various components from propolis. *Molecular Nutrition & Food Research*.

[7] Xie F., Su M., Qiu W. (2013). Kaempferol promotes apoptosis in human bladder cancer cells by inducing the tumor suppressor, PTEN. *International Journal of Molecular Sciences*.

[8] Wu X., Obata T., Khan Q., Highshaw R. A., de Vere White R., Sweeney C. (2004). The phosphatidylinositol‐3 kinase pathway regulates bladder cancer cell invasion. *BJU International*.

[9] Halimah E., Diantini A., Destiani D. P. (2015). Induction of caspase cascade pathway by kaempferol-3-O-rhamnoside in LNCaP prostate cancer cell lines. *Biomedical Reports*.

[10] Xu D., Lin T. H., Li S. (2012). Cryptotanshinone suppresses androgen receptor-mediated growth in androgen dependent and castration resistant prostate cancer cells. *Cancer Letters*.

[11] Xu D., Chen Q., Liu Y., Wen X. (2017). Baicalein suppresses the androgen receptor (AR)-mediated prostate cancer progression *via* inhibiting the AR N-C dimerization and AR-coactivators interaction. *Oncotarget*.

[12] Ni J., Pang S. T., Yeh S. (2007). Differential retention of *α*‐vitamin E is correlated with its transporter gene expression and growth inhibition efficacy in prostate cancer cells. *The Prostate*.

[13] Kuo W. T., Tsai Y. C., Wu H. C. (2015). Radiosensitization of non-small cell lung cancer by kaempferol. *Oncology Reports*.

[14] Song H., Bao J., Wei Y. (2015). Kaempferol inhibits gastric cancer tumor growth: an in vitro and in vivo study. *Oncology Reports*.

[15] Lee J., Kim J. H. (2016). Kaempferol inhibits pancreatic cancer cell growth and migration through the blockade of EGFR-related pathway in vitro. *PLoS One*.

[16] Kim S. H., Hwang K. A., Choi K. C. (2016). Treatment with kaempferol suppresses breast cancer cell growth caused by estrogen and triclosan in cellular and xenograft breast cancer models. *The Journal of Nutritional Biochemistry*.

[17] Luo H., Rankin G. O., Li Z., Depriest L., Chen Y. C. (2011). Kaempferol induces apoptosis in ovarian cancer cells through activating p53 in the intrinsic pathway. *Food Chemistry*.

[18] Dang Q., Song W., Xu D. (2015). Kaempferol suppresses bladder cancer tumor growth by inhibiting cell proliferation and inducing apoptosis. *Molecular Carcinogenesis*.

[19] Ahmadi S. A., Moinfar M., Gohari Moghaddam K., Bahadori M. (2010). Practical application of angiogenesis and vasculogenic mimicry in prostatic adenocarcinoma. *Archives of Iranian Medicine*.

[20] Bhattacharyya S., Mitra D., Ray S. (2019). Reversing effect of Lupeol on vasculogenic mimicry in murine melanoma progression. *Microvascular Research*.

[21] Lin X., Sun R., Zhao X. (2017). C-myc overexpression drives melanoma metastasis by promoting vasculogenic mimicry via c-myc/snail/Bax signaling. *Journal of Molecular Medicine*.

[22] Pastorino O., Gentile M. T., Mancini A. (2019). Histone deacetylase inhibitors impair vasculogenic mimicry from glioblastoma cells. *Cancers*.

[23] Xiao Y., Cheng L., Xie H. J. (2018). Vinorelbine cationic liposomes modified with wheat germ agglutinin for inhibiting tumor metastasis in treatment of brain glioma. *Artificial Cells, Nanomedicine, and Biotechnology*.

[24] Shen R., Wu T., Huang P., Shao Q., Chen M. (2019). The clinicopathological significance of ubiquitin-conjugating enzyme E2C, leucine-rich repeated-containing G protein-coupled receptor, WW domain-containing oxidoreductase, and vasculogenic mimicry in invasive breast carcinoma. *Medicine*.

[25] Ou H., Chen Z., Xiang L. (2019). Frizzled 2‐induced epithelial‐mesenchymal transition correlates with vasculogenic mimicry, stemness, and Hippo signaling in hepatocellular carcinoma. *Cancer Science*.

[26] Wang H., Huang B., Li B. M. (2018). ZEB1‐mediated vasculogenic mimicry formation associates with epithelial–mesenchymal transition and cancer stem cell phenotypes in prostate cancer. *Journal of Cellular and Molecular Medicine*.

[27] Liu R., Yang K., Meng C., Zhang Z., Xu Y. (2012). Vasculogenic mimicry is a marker of poor prognosis in prostate cancer. *Cancer Biology & Therapy*.

[28] Yeo C., Lee H. J., Lee E. O. (2019). Serum promotes vasculogenic mimicry through the EphA2/VE-cadherin/AKT pathway in PC-3 human prostate cancer cells. *Life Sciences*.

[29] Le Marchand L., Murphy S. P., Hankin J. H., Wilkens L. R., Kolonel L. N. (2000). Intake of flavonoids and lung cancer. *Journal of the National Cancer Institute*.

[30] Kim B. W., Lee E. R., Min H. M. (2008). Sustained ERK activation is involved in the kaempferol-induced apoptosis of breast cancer cells and is more evident under 3-D culture condition. *Cancer Biology & Therapy*.

[31] Kang G. Y., Lee E. R., Kim J. H. (2009). Downregulation of PLK-1 expression in kaempferol-induced apoptosis of MCF-7 cells. *European Journal of Pharmacology*.

[32] Bobe G., Albert P. S., Sansbury L. B. (2010). Interleukin-6 as a potential indicator for prevention of high-risk adenoma recurrence by dietary flavonols in the polyp prevention trial. *Cancer Prevention Research*.

[33] Qazi B. S., Tang K., Qazi A. (2011). Recent advances in underlying pathologies provide insight into interleukin-8 expression-mediated inflammation and angiogenesis. *International Journal of Inflammation*.

[34] Luo H., Rankin G. O., Juliano N., Jiang B. H., Chen Y. C. (2012). Kaempferol inhibits VEGF expression and in vitro angiogenesis through a novel ERK-NF *κ*B-cMyc-p21 pathway. *Food Chemistry*.

