# Supplementary material for: Kaempferol Promotes Apoptosis While Inhibiting Cell Proliferation via Androgen-Dependent Pathway and Suppressing Vasculogenic Mimicry and Invasion in Prostate Cancer
Source: Anal Cell Pathol (Amst). 2019 Dec 1;2019:1907698. doi: 10.1155/2019/1907698 (PMC6913338; doi:10.1155/2019/1907698)
Supplement: Supplementary Materials — (A) Dihydrotestosterone (DHT) which is an androgen. (B) Kaempferol, a natural flavonol widely found from fruits and vegetables. (C) Cryptotanshinone, a major tanshinone isolated from Salvia miltiorrhiza and exhibits multiple activities. (D) Baicalein, a flavonoid originally isolated from the roots of Scutellaria baicalensis Georgi. The structure of kaempferol, Cryptotanshinone, or Baicalein is similar to DHT. [file 1907698.f1.pdf]

Supplementary figure

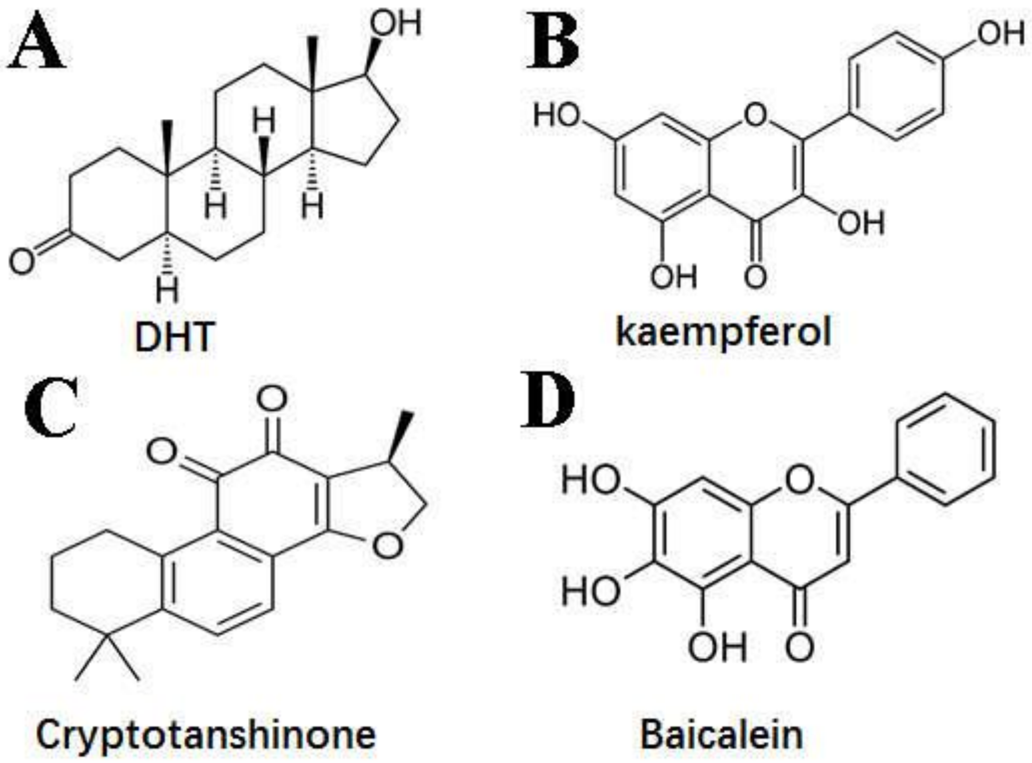

(A) Dihydrotestosterone (DHT) which is an androgen. (B) kaempferol, a natural flavonol widely found from fruits and vegetables.(C) Cryptotanshinone, a major tanshinone isolated from Salvia miltiorrhiza and exhibits multiple activities.(D) Baicalein, a flavonoid originally isolated from the roots of Scutellaria baicalensis Georgi. The structure of kaempferol, Cryptotanshinone or Baicalein is similar to DHT.
